# Financial Disclosures Reported by Industry Among Authors of the American Academy of Ophthalmology Clinical Practice Guidelines

**DOI:** 10.1001/jamaophthalmol.2023.0267

**Published:** 2023-03-16

**Authors:** Anne Xuan-Lan Nguyen, Maxine Joly-Chevrier, David-Dan Nguyen, Albert Y. Wu

**Affiliations:** 1 Faculty of Medicine and Health Sciences, McGill University, Montreal, Quebec, Canada; 2 Faculty of Medicine, University of Montreal, Montreal, Quebec, Canada; 3 Division of Urology, Department of Surgery, University of Toronto, Toronto, Ontario, Canada; 4 Department of Ophthalmology, Stanford University School of Medicine, Stanford, California

## Abstract

**Importance:**

Recommendations of clinical guidelines affect physicians’ care delivery. Potential bias and undeclared conflicts of interests (COIs) among guideline authors can impact clinical practice decisions.

**Objective:**

To assess financial disclosures reported by physician authors of the American Academy of Ophthalmology (AAO) Practice Pattern Guidelines compared with those reported by industry to evaluate the disclosures’ accuracy.

**Design, Setting, and Participants:**

In this cross-sectional study, all clinical guidelines in the AAO Preferred Practice Patterns (PPP) since 2013 (first year with publicly available industry payment reports) were reviewed on May 1, 2022. Guideline physician authors’ name and their reported COI disclosure were extracted from the guideline publication. Payments to physician authors reported by industry were retrieved from the US Centers for Medicare & Medicaid Open Payments database. Physician authors serving on the AAO guideline committee were included.

**Main Outcomes and Measures:**

The primary outcome measure was the accuracy of authors’ COIs disclosure. Secondary outcome measures were payments to physician authors reported by industry, the types of payments, and authors’ gender.

**Results:**

A total of 24 AAO guidelines released between 2016 and 2020 were included. Per guideline, there was a mean (SD) of 7.83 (2.24) physician authors. After removing 14 nonphysician authors, 188 physician author names remained, including 83 names assigned as women (44.1%) and 105 names assigned as men (55.9%). Authors could be counted multiple times in these 188 names. According to the Open Payments database, industry reported that 112 of 188 physician authors (59.6%) had at least received 1 payment while serving on the guideline committee, with a payment mean (SD) of $29 849.35 ($54 131.56). According to AAO guidelines, 149 authors (79.3%) had no financial disclosures while serving on the guideline committee. Among these 149 authors, most authors (81 [54.4%]) had payments reported by industry on the Open Payments database not disclosed within the guideline reports. Women physicians were paid significantly more than men for total payments (median [IQR] payments, $15 265 [$598.47-$41 104.67] vs $301.48 [$218.85-$14 615.09]; difference, $14 963.52; *P* = .003).

**Conclusions and Relevance:**

Industry reported physician guideline authors to have received significant industry payments, some of which were not disclosed within information of the guidelines. To strengthen author transparency regarding these reported disclosures, the authors may want to review and resolve such potential discrepancies during the review and subsequent publication of guidelines.

## Key Points

**Question:**

What conflicts of interests or financial disclosures are reported by physician guideline authors serving on guideline committees of the American Association of Ophthalmology (AAO) relative to payments reported by industry on the US Centers for Medicare & Medicaid Open Payments database?

**Findings:**

In this cross-sectional study, among 149 physician guideline authors who declared having no financial disclosures while serving on the AAO guideline committee, 81 physician authors (54.4%) had payments indicated by industry on the Open Payments database not reported within the guidelines.

**Meaning:**

Discordance in disclosures of physician authors serving on AAO guideline committees should be evaluated to strive for accurate disclosures.

## Teaser

This cross-sectional study assesses financial disclosures reported by physician authors of the American Academy of Ophthalmology (AAO) Practice Pattern Guidelines compared with those reported by industry to evaluate the disclosures’ accuracy.

## Introduction

Rooted in evidence-based medicine, clinical practice guidelines (CPGs) use available scientific data to provide the latest recommendations with the goal of optimizing patient care.^1^ Guidelines can be subject to bias and conflicts of interest (COIs), which impact recommendations.^2^ Since the Physician Payments Sunshine Provision of the Affordable Care Act in 2013, medical supply manufacturers (ie, industry) must report specific payments to physicians and medical teaching institutions. Some companies also need to report their physicians’ ownership and investment interests.^3^ To ensure transparency, the information is centralized on the US Centers for Medicare & Medicaid Open Payments database, a public website.^4^

Financial compensation can compromise medical research by skewing study outcomes and physicians’ clinical decisions.^5,6^ Readers should thus be aware of physician authors’ potential COIs. To our knowledge, the extent to which COIs systematically bias the field of ophthalmology by influencing society guidelines and the accuracy of COIs reported by authors of guidelines or reported by industry to have been given to those authors is unknown.

Considering this gap in the ophthalmology literature, we assessed payments reported by authors of the American Academy of Ophthalmology (AAO) Practice Pattern Guidelines compared with payments reported by industry to have been given to these authors to evaluate the disclosures’ accuracy and authors’ compliance to the Council of Medical Specialty Societies’ Code for Interactions with Companies.^7^ We further determined author gender (social construct, assigned using an application program interface) to evaluate potential differences in COIs.

## Methods

### AAO Clinical Guideline Selection

All authors reviewed all clinical guidelines in the Preferred Practice Patterns (PPP) section of the AAO website^8^ and reviewed available industry payment data listed from the Open Payments database^4^ on May 1, 2022. The study period was determined based on publicly available industry payment data only being reported as of 2013. Indeed, the national policies mandating Open Payments were originally enacted in 2013 with the goal of collecting and publishing information about payments reporting entities made to covered recipients.^9^ This study was exempt from research ethics board review per Article 2.2 of the Tri-Council Policy Statement. The described research adhered to the tenets of the Declaration of Helsinki. This study followed the Strengthening the Reporting of Observational Studies in Epidemiology (STROBE) reporting guideline.

### Data Extraction

Two authors (A. X.-L. N. and M. J.-C.) retrieved guideline authors’ names and their reported COI disclosures on the guideline publication. Guideline authors only included guideline writers. Nonphysician guideline authors were excluded. We further documented if the authors were chairs or cochairs of the AAO subspecialty guidelines committee. The exact dates in which physician authors joined AAO’s guidelines committees were unknown. Physician authors were counted multiple times if they authored more than 1 AAO guideline.

Three authors (A. X.-L. N., M. J.-C., and D.-D. N.) entered eligible authors’ names into the Open Payments search tool and cross-matched their full name, role (allopathic and osteopathic physicians), medical specialty (ophthalmology), and location to identify the correct individuals and extract their payment data matching the disclosure period indicated on the AAO guideline. Indeed, the AAO guidelines provide specific disclosure periods by indicating months and years. For example, if a guideline has a disclosure period from January to October 2019, we matched the same time period on Open Payments.

Payment data were categorized into general payments, research payments, research funding, and ownerships. General payments are payments not associated with research studies, contrary to research payments. Examples of general payments include consulting and speaking fees, honoraria, gifts, and royalties not related to research studies. Associated research funding is defined as funding received for a research project where the physician is named as principal investigator. Ownership payments consist of the actual dollar amount invested and the value of the ownership or payment investment interest.^9^ One of us (A. X.-L. N.) determined physician authors’ gender (social construct) by inputting their first names into Gender API,^10^ which is an application program interface assigning gender with 98% accuracy.^11^

### Statistical Analysis

Descriptive statistics were calculated using Stata/IC version 16.1 (StataCorp). The Kruskal-Wallis test by ranks was performed to test whether there was a significant difference in median total payments between men and women. *P* values were not adjusted for multiple analyses. *P* values less than .05 were considered statistically significant, and all *P* values were 2-tailed.

## Results

### Guideline Characteristics and Guideline Author Demographic Characteristics

A total of 24 guidelines released between 2016 and 2020 by the AAO were included. PPPs were divided into subspecialty (Table 1), including 7 focused on retinal pathologies, 6 on corneal and external diseases, 4 on adult strabismus and pediatrics, 3 on glaucoma, 1 on low vision, 1 on optics, 1 on cataract, and 1 on comprehensive ophthalmology. Per guideline, there was a mean (SD) of 7.83 (2.24) physician authors.

**Table 1.** Summary of American Academy of Ophthalmology (AAO) Guidelines in the Preferred Practice Patterns (PPP) Section

Guideline/PPP	AAO Writing Committee	Physician authors, No.			Subspecialty
		Physician authors (total authors)	With financial disclosures on PPP	With financial disclosures reported by industry on Open Payments	
1. Primary Open-Angle Glaucoma Suspect 2020	Glaucoma PPP Panel 2019-2020	8	3	3	Glaucoma
2. Comprehensive Adult Medical Eye Evaluation 2020	PPPs Committee 2020	7 (8)	2	4	Comprehensive
3. Primary Angle-Closure Disease 2020	Glaucoma PPP Panel 2019-2020	8	2	3	Glaucoma
4. Primary Open-Angle Glaucoma 2020	Glaucoma PPP Panel 2019-2020	8	3	3	Glaucoma
5. Diabetic Retinopathy 2019	Retina/Vitreous PPP Panel 2018-2019	7	1	4	Retina/vitreous
6. Age-Related Macular Degeneration 2019	Retina/Vitreous PPP Panel 2018-2019	7	1	4	Retina/vitreous
7. Retinal Vein Occlusions 2019	Retina/Vitreous PPP Panel 2018-2019	7	1	4	Retina/vitreous
8. Idiopathic Macular Hole 2019	Retina/Vitreous PPP Panel 2018-2019	7	1	4	Retina/vitreous
9. Idiopathic Epiretinal Membrane and Vitreomacular Traction 2019	Retina/Vitreous PPP Panel 2018-2019	7	1	4	Retina/vitreous
10. Retinal and Ophthalmic Artery Occlusions 2019	Retina/Vitreous PPP Panel 2018-2019	7	1	4	Retina/vitreous
11. Adult Strabismus 2019	Adult Strabismus PPP Panel 2017-2019	17 (18)	4	4	Adult strabismus
12. Posterior Vitreous Detachment, Retinal Breaks, and Lattice Degeneration 2019	Retina/Vitreous PPP Panel 2018-2019	7	1	4	Retina/vitreous
13. Dry Eye Syndrome 2018	Cornea/External Disease PPP Panel 2017-2018	9 (10)	3	9	CED
14. Blepharitis 2018	Cornea/External Disease PPP Panel 2017-2018	9 (10)	3	9	CED
15. Corneal Ectasia 2018	Cornea/External Disease PPP Panel 2017-2018	9 (10)	0	9	CED
16. Conjunctivitis 2018	Cornea/External Disease PPP Panel 2017-2018	9 (10)	3	9	CED
17. Corneal Edema and Opacification 2018	Cornea/External Disease PPP Panel 2017-2018	9 (10)	3	9	CED
18. Bacterial Keratitis 2018	Cornea/External Disease PPP Panel 2017-2018	9 (10)	3	9	CED
19. Vision Rehabilitation 2017	Vision Rehabilitation Committee 2016-2017	7 (8)	0	1	Vision rehabilitation
20. Refractive Errors & Refractive Surgery 2017	Refractive Management/Intervention PPP 2016-2017	6 (7)	0	4	Refractive errors/surgery
21. Esotropia and Exotropia 2017	Pediatric Ophthalmology/Strabismus PPP Panel 2016-2017	6 (7)	0	1	Pediatric/strabismus
22. Pediatric Eye Evaluations 2017	Pediatric Ophthalmology/Strabismus PPP Panel 2016-2017	6 (7)	0	1	Pediatric/strabismus
23. Amblyopia 2017	Pediatric Ophthalmology/Strabismus PPP Panel 2016-2017	6 (7)	0	1	Pediatric/strabismus
24. Cataract in the Adult Eye 2016	AAO PPP Cataract/Anterior Segment Panel, Hoskins Center for Quality Eye Care	6 (7)	3	5	Cataract/anterior segment

Abbreviation: CED, cornea/external disease.

There were 14 nonphysician author names, including 2 assigned as women and 12 assigned as men. These nonphysician authors wrote between 1 and 7 guidelines each and held one of the following degrees: CO, COMT, JD, MHS, MPH, PhD, or ScM. After excluding 14 nonphysician author names, 188 author names remained, including 83 assigned as women (44.1%) and 105 assigned as men (55.9%). These author names represented 66 different authors, as authors wrote between 1 and 8 guidelines each. According to the AAO guidelines, 149 guideline authors (79.3%) had no financial disclosures while serving on the AAO guideline committee.

### Industry Payments Reported for Guideline Authors

Among the 149 guideline authors who reported having no financial disclosures, 81 (54.4%) had payments reported by industry on the Open Payments database while serving on the AAO guideline committee. More specifically, guideline authors with no financial disclosures reported on the guidelines had the following payments reported by industry on the Open Payments Database during the AAO disclosure period: a median (IQR; range) of 5 (3-8; 1-115) payments and a mean (SD) of 8.2 (13.8) payments, totaling between $9.78 and $79 602.07 (median [IQR] total, $332.17 [$199.03-$14 615.09]; mean [SD] total, $10 768.05 [$28 676.47]). A total of 16 guideline authors (19.8%) had payments of less than $100 reported by industry on the Open Payments database.

According to the Open Payments database, 112 of 188 physician authors (59.6%) had been reported by industry to have received at least 1 payment while serving on the AAO guideline committee (Table 1; Figure). Among them, there were 61 assigned as women (54.5%) and 51 assigned as men (45.5%). Physician authors had been reported by industry to have received a total of $3 343 127.48 in general payments and associated research fundings, including $2 541 227.78 to women physician authors and $801 899.70 to men. None of the physician authors had been reported by industry to have received research payments nor ownership payments. Physician authors had been reported by industry to have received a mean (SD) of $29 849.35 ($54 131.56), with total payments ranging from $9.78 to $225 958. The 98 physician authors with general payments had been reported by industry to have received a mean (SD) of $22 770.49 ($51 732.14), with total general payments ranging from $9.78 to $207 658. The 42 physician authors with associated research fundings had been reported by industry to have received a mean (SD) of $26 467.12 ($19 328.85), with total associated research fundings ranging from $265.20 to $57 572.20.

**Figure.**
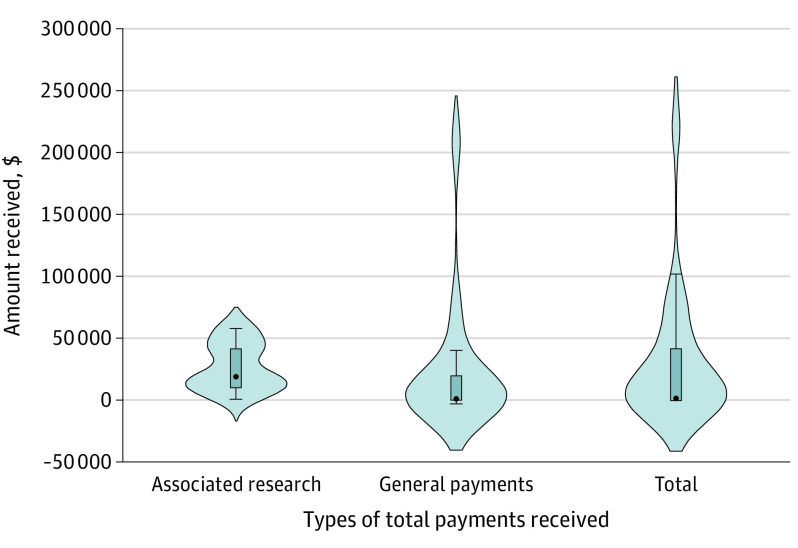
Total Amount of Payments Reported by Industry to Have Been Received by American Academy of Ophthalmology Clinical Practice Guideline Authors Sorted by Payment Types The point represents the median, and the box represents the IQR. The distance between the lower and the upper whiskers is equal to 1.5-fold the IQR. The shaded area shows the probability density of the data at different values. The shaded area is further smoothed by a kernel density estimator.

As indicated in Table 2, physician authors had been reported by industry to have received a mean (SD) of $29 849.35 ($54 131.56) and a median (IQR) of $691.17 ($218.85-$41 104.67) in total payments. Women physician authors had been reported by industry to have received a mean (SD) of $41 659.47 ($66 364.94) and a median (IQR) of $15 265 ($598.47-$41 104.67) in payments. Men physician authors had been reported by industry to have received a mean (SD) of $15 723.52 ($29 090.22) and a median (IQR) of $301.48 ($218.85-$14 615.09) in payments. Women were therefore reported by industry to have been paid more than men, with a difference in medians of $14 963.52 (95% CI, 0.059-0.281; *P* = .003).

**Table 2.** Total Payments Reported by Industry on Open Payments

Gender	Total, No.	Median (IQR), $	Difference in medians (95% CI)^a^	*P* value^b^
Total payments	112	691.17 (218.85-41 104.67)	NA	NA
Women	61	15265 (598.47-41 104.67)	14 963.52 (0.059-0.281)	.003
Men	51	301.48 (218.85-14 615.09)		
General payments	98	465.32 (166.84-18 768.07)	NA	NA
Women	53	691.17 (199.03-18 768.07)	389.69 (−0.057 to 0.189)	.29
Men	45	301.48 (83.78-10 337.88)		
Associated research fundings		18 300 (9400.29-41 028.52)	NA	NA
Women	28	29 664.26 (15 265-49 300.36)	20 263.97 (0.140-0.521)	<.001
Men	14	9400.29 (6000-9400.29)		

Abbreviation: NA, not applicable.

^a^ Obtained using Somers D.

^b^ Obtained using the Kruskal-Wallis rank test.

### Guidelines Chairs and Cochairs

There was a total of 30 physician authors serving as chairs and cochairs in the AAO guidelines reviewed, with 6 guidelines cochaired by 2 physicians. A total of 6 of 30 chairs and cochairs (20%) reported financial disclosures in the AAO guidelines. Of these, 21 chairs and cochairs had financial disclosures reported by industry on the Open Payments database, with 1 to 115 payments (median [IQR] payments, 5 [3-115]; mean [SD] payments, 35.76 [51.38]) totaling $61.56 to $79 602.07 (median [IQR] of $15 265 [$301.48-$79 602.07]; mean [SD] of $27 689.74 [$34 222.31]).

## Discussion

Some ophthalmologists are reported by industry to receive substantial payments from industry on the Open Payments database.^5^ The payments reported by industry to have been received by physician authors of AAO guidelines was substantial, with a median (IQR) of $691.17 ($218.85-$41 104.67) and a mean (SD) of $29 849.35 ($54 131.56), which is higher compared with industry payments received by clinical guideline physician authors in other medical specialties.^12,13,14^ All authors reviewed all AAO guidelines published from 2013 until May 2022, and 2 authors (A. X.-L. N. and M. J.-C.) extracted physician guideline authors’ COIs. Three authors (A. X.-L. N., M. J.-C., and D.-D. N.) retrieved physician payments reported by industry using the Open Payments database.

AAO’s policy statement stipulated that committee members must disclose all financial relationships with companies.^15^ However, more than half of physician authors (81 [54.4%]) who declared having no financial disclosures while serving on the AAO guideline committee had payments reported by industry on the Open Payments database. Studies in other medical specialties that examined physicians who authored clinical guidelines of leading specialty organizations similarly reported a disconnect between the COIs reported in the guidelines and those reported by industry in Open Payment systems.^2,12,13,14^ If truly representing errors, this disconnect between COIs reported in guidelines and in Open Payments may contribute to potential biases in guidelines of national medical organizations, including the AAO.^2^ Furthermore, the Institutes of Medicine Guidelines for trustworthy CPGs recommends that 50% or more authors on CPG committees have no COIs.^16^ While nominally all the AAO guidelines fit this criterion, our analyses show that 16 of 24 guidelines now fail and may be considered untrustworthy. Additionally, these best practice guidelines for CPGs recommend that committee chairs have no COIs, which is not the case in 6 of 30 chairs and cochairs based on the AAO guidelines’ own disclosures and in 21 of these same chairs and cochairs based on the information reported by industry on the Open Payments database.

Among the 112 physician authors who were reported by industry to have received at least 1 industry payment, women (54.5%) were more represented than men. Women physicians were reported by industry to have been paid significantly more than men for total payments (difference, $14 963.52; 95% CI, 0.059-0.281; *P* = .003). Our findings differ from prior studies reporting that women physicians were underrepresented in industry compensation and paid less in industry partnerships compared with men.^17,18^ Extended research should be conducted to understand these financial differences.

### Limitations

Our study presents limitations. The first limitation is that we are using the Open Payments database. Therefore, we are relying on payments reported by industry, which do not necessarily indicate payments truly received by physicians. Indeed, Open Payments could have reporting errors from a company; some listings in Open Payments may have gone to a physician’s employer or a payment declined by a physician still could be reported in Open Payments system. Second, the SDs calculated for the payments reported to have been received indicate that the data are quite skewed, as they are 2-fold greater than the means. It is also not possible to fully assess ophthalmologist’s financial disclosures due to the limits of the Physician Payment Sunshine Act. The Sunshine Act only requires payment reporting of companies that sell products covered by government programs and that offer compensation worth more than $10.^5^ However, even small compensations as low as $10 may influence physicians. It was reported that there was an association between sponsored meals by pharmaceutical companies and the increase in their drug prescriptions.^5^ Our findings may underestimate the effect of COIs on guidelines authors. Additionally, some genders may be inaccurate since we assigned gender (social construct) using Gender API. Gender API does not capture the spectrum of gender identities, which limits genders included in this study.

## Conclusions

Moving forward, AAO policies could be modified by reinforcing authors’ reviews of their disclosures with disclosures reported by industry on the Open Payments database. Increasing authors’ awareness and understanding of the AAO policy statement may also contribute to better declaration of COIs in clinical guidelines. It remains unclear if COI reporting alone is sufficient to mitigate industry influence on evidence-based guidelines.^19^

## Notes

Supplement. Data Sharing Statement
