# Supplementary material for: Financial Disclosures Reported by Industry Among Authors of the American Academy of Ophthalmology Clinical Practice Guidelines
Source: JAMA Ophthalmol. 2023 Mar 16;141(4):378–83. doi: 10.1001/jamaophthalmol.2023.0267 (PMC10020930; doi:10.1001/jamaophthalmol.2023.0267)
Supplement: Supplement. — Data Sharing Statement [file jamaophthalmol-e230267-s001.pdf]

Data Sharing Statement

Nguyen. Financial Disclosures Reported by Industry Among Authors of the American Academy of Ophthalmology Clinical Practice Guidelines. *JAMA Ophthalmol*. Published March 16, 2023. doi:10.1001/jamaophthalmol.2023.0267

Data
Data available: Yes
Data types: Data (not involving human participants)
How to access data: Data will be shared upon request.
When available: With publication

Supporting Documents
Document types: None

Additional Information
Who can access the data: Data will be shared upon request.
Types of analyses: For specified purposes
Mechanisms of data availability: With investigator support
